# Diet, physical activity, and sleep in relation to postprandial glucose responses under free-living conditions: an intensive longitudinal observational study

**DOI:** 10.1186/s12966-024-01693-5

**Published:** 2024-12-18

**Authors:** Jiali Yao, Victoria K. Brugger, Sarah M. Edney, E-Shyong Tai, Xueling Sim, Falk Müller-Riemenschneider, Rob M. van Dam

**Affiliations:** 1 Saw Swee Hock School of Public Health, National University of Singapore, Singapore, Singapore; 2 Epidemiology, Biostatistics and Prevention Institute, University of Zurich, Zurich, Switzerland; 3 Yong Loo Lin School of Medicine, National University of Singapore, Singapore, Singapore; 4 Digital Health Center, Berlin Institute of Health, Charité-Universitätsmedizin Berlin, Berlin, Germany; 5 Department of Exercise and Nutrition Sciences, Milken Institute School of Public Health, The George Washington University, Washington, DC USA; 6 Department of Nutrition, Harvard T.H. Chan School of Public Health, Boston, MA USA

**Keywords:** Continuous glucose monitoring, Postprandial glucose response, Nutrition, Physical activity, Sleep, Mobile health, Free-living

## Abstract

**Background:**

It remains unclear what lifestyle behaviors are optimal for controlling postprandial glucose responses under real-world circumstances in persons without diabetes. We aimed to assess associations of diet, physical activity, and sleep with postprandial glucose responses in Asian adults without diabetes under free-living conditions.

**Methods:**

We conducted an observational study collecting intensive longitudinal data using smartphone-based ecological momentary assessments, accelerometers, and continuous glucose monitors over nine free-living days in Singaporean men and women aged 21–69 years without diabetes. The outcome was the 2-h postprandial glucose incremental area under the curve (mmol/l*min). Associations were estimated using linear mixed-effect models.

**Results:**

The analyses included 11,333 meals in 789 participants. Greater variations in glucose and lifestyle measures were observed within individuals than between individuals. Higher consumption of carbohydrate-rich and deep-fried foods and lower consumption of protein-rich foods were significantly associated with higher postprandial glucose levels (incremental area under the curve). The strongest association was observed for including refined grains (46.2 [95% CI: 40.3, 52.1]) in meals. Longer postprandial light-intensity physical activity (-24.7 [(-39.5, -9.9] per h) and moderate-to-vigorous-intensity physical activity (-58.0 [-73.8, -42.3]) were associated with substantially lower postprandial glucose levels. Longer daily light-intensity physical activity (-7.5 [-10.7, -4.2]) and sleep duration (-2.7 [-4.4, -1.0]) were also associated with lower postprandial glucose levels. Furthermore, postprandial glucose levels were the lowest in the morning and the highest in the afternoon. The results were largely consistent for males and females and for participants with and without prediabetes.

**Conclusions:**

Consuming less refined grains and more protein-rich foods, getting more physical activity (particularly during the postprandial period), and having a longer sleep duration were associated with lower postprandial glucose levels in Asian adults without diabetes. Our findings support multi-component lifestyle modifications for postprandial glucose control and highlight the importance of the timing of eating and physical activity.

**Supplementary Information:**

The online version contains supplementary material available at 10.1186/s12966-024-01693-5.

## Background

Postprandial glucose levels, the blood glucose concentrations following food intake, are important indicators of glycemic control and overall cardiometabolic health [1, 2]. Postprandial hyperglycemia is characterized by abnormally rapid and large spikes in postprandial glucose levels and is among the earliest signs of metabolic deterioration [3]. Moreover, postprandial hyperglycemia is associated with a higher risk of cardiovascular diseases in both persons with and without diabetes, independent of fasting glucose and HbA1c levels [1, 4].

Lifestyle behaviors, including diet, physical activity, and sleep, play essential roles in glycemic control. A growing number of studies have demonstrated the promise of healthy lifestyle behaviors to improve acute postprandial glucose responses [1, 5]. However, existing evidence is skewed towards individuals with diabetes and mostly originated from small laboratory-based studies or studies that relied on standardized meals. Large studies examining diet, physical activity, and sleep simultaneously using high-resolution objective or real-time data are also lacking, especially in Asian populations. In addition, limited by the collection of data at a single time point, previous studies largely focused on stable between-person differences at a population level. Effects of temporal within-person lifestyle variations on postprandial glucose responses remain unclear. Longitudinal data collected at multiple time points allow simultaneous examination of associations at between-person and within-person levels. Analysis at the between-person level relates variation in usual levels of lifestyle behaviors to variation in postprandial glucose responses between individuals. In contrast, analysis at the within-person level relates temporal variation in lifestyle behaviors to variation in postprandial glucose levels in the same individual [6].

To better understand optimal lifestyle behaviors for postprandial glucose control in real-world settings, we conducted a study with intensive longitudinal measures using smartphone app-based ecological momentary assessments (EMA), accelerometers, and continuous glucose monitors (CGM) over nine days in Singapore adults under free-living conditions. We investigated associations between lifestyle factors (i.e., diet, physical activity, and sleep) and postprandial glucose responses to free-choice meals at between-person and within-person levels.

## Methods

### Study design and participants

**Fig. 1:**
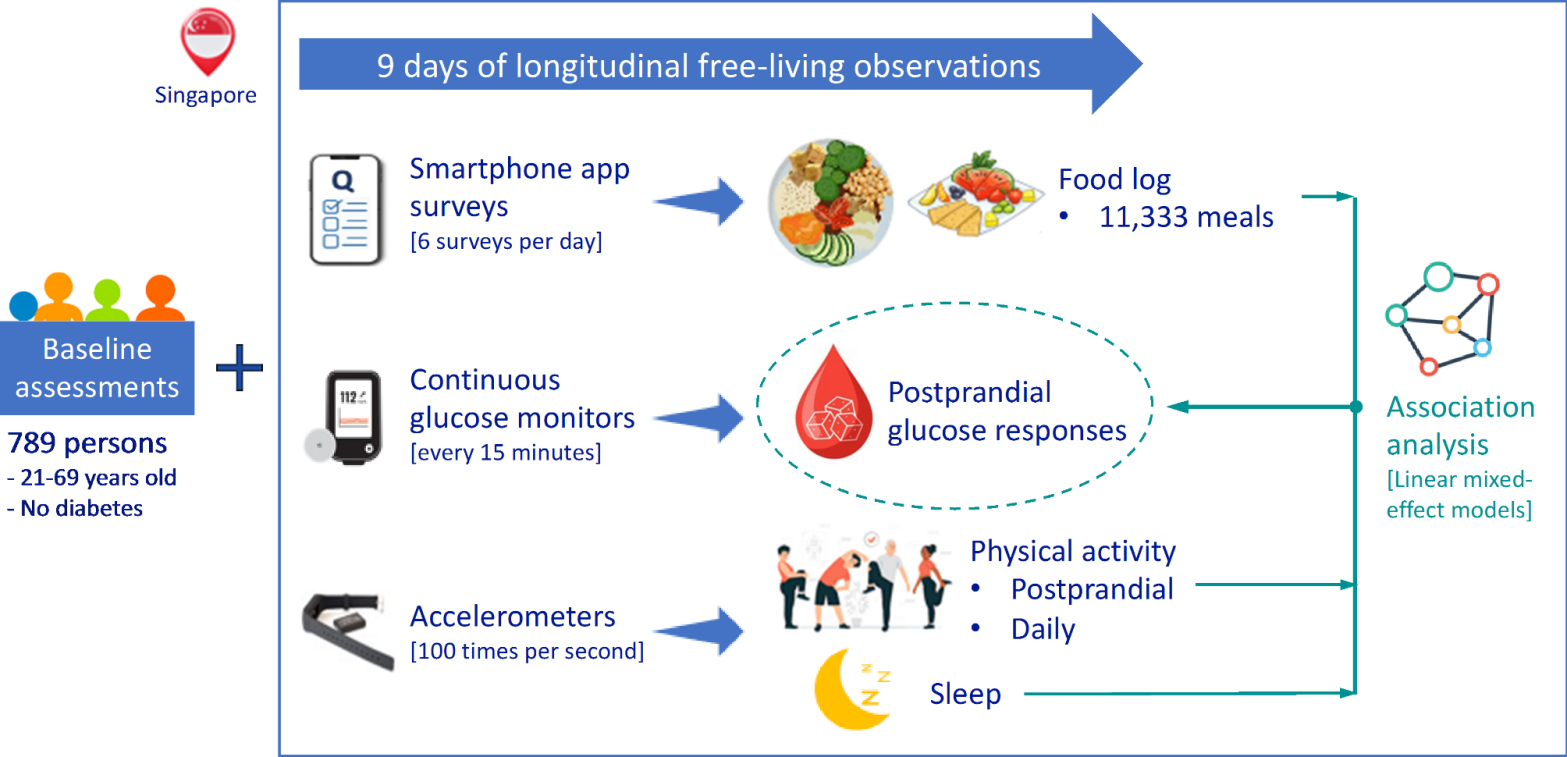
Overview of study design

The Continuous Observations of Behavioral Risk Factors in Asia (COBRA) is a longitudinal observational study. Details of COBRA have been published elsewhere [7]. In brief, COBRA recruited Singapore adults aged 21 to 69 years of Chinese, Malay, or Indian ethnicity. Participants were excluded if they had a known sensitivity to medical-grade adhesive, bleeding disorder, severe mental health condition, or a history of cardiovascular diseases, diabetes, cancer, kidney failure, or thyroid diseases. Following the baseline visit with a questionnaire interview and physical examination, COBRA participants underwent nine days of continuous diet- and movement-related monitoring in free-living conditions using multiple ‘real-time’ data collection methods, including smartphone app-based EMA surveys, accelerometers, and CGMs (an overview in Fig. 1).

This study used data from 820 participants without diabetes recruited between May 2021 and April 2024 with CGM data (details in Supplementary **Methods)**. All participants provided informed consent. Ethical approval was obtained from the Institutional Review Board of the National University of Singapore (NUS ref: NUS-IRB-2020-50).

### Food logging via EMA surveys

Participants were instructed to log all their food intakes via EMA surveys. An app (Ethica; Avicenna Research Inc) was installed on participants’ smartphones at the baseline visit to deliver six EMA surveys per day during the nine free-living days. The six EMA surveys were sent at random times within six time windows across the day (i.e., 8:00–9:30, 10:30 − 12:00, 13:00–14:30, 15:30 − 17:00, 18:00–19:30, and 20:30 − 21:30 h). Participants received up to four reminders spaced 10 min apart for each of the first five EMA surveys and up to two reminders spaced 10 min apart for the last EMA survey. The reminders were sent via app push notifications before participants responded to the survey.

The first EMA survey of the day asked participants to report food intake, if any, for two occasions: one for intake after the last EMA survey and before sleep yesterday (applicable to the 2nd to 9th day only) and one for food intake since waking up. The other five EMA surveys asked about any food intake since the last EMA survey. Participants reported meal time in ‘HH: MM’ format, which was integrated with the automatically recorded EMA response date to form the full meal timestamp. For meals taken after the last EMA and before sleep yesterday with a meal time before midnight, the meal date was the day before EMA response date. For the other meals, the meal date was the EMA response date. Given our EMA design with a high sampling rate, we assumed participants had at most one meal and had one meal time to report on each occasion. Participants also recorded their post-meal satiety level using a 6-point scale, with 1 referring to ‘still hungry’ and 6 referring to ‘extremely full’. To reduce participant burden and improve response rates, we did not ask for detailed information on the consumed foods (e.g., portion size information). Instead, we provided three pre-defined Singapore-specific food group lists for participants to select the food groups eaten using three check-all-that-apply questions (details in Supplementary **Methods**).

### Physical activity and sleep measures via accelerometers

During the nine free-living days, we instructed participants to wear an accelerometer (AX3; Axivity Ltd) on the non-dominant wrist at all times to collect movement behavior at a frequency of 100 Hz. The paired Axivity Wrist Band was used to fit AX3 following the recommended mounting convention, with the arrow mark on the wristband aligned with the arrow on AX3 [8]. Raw accelerometer data were processed using R package GGIR (version 3.1-1) to derive durations of physical activity and sleep specific to each meal, including light-intensity physical activity (LPA) and moderate-to-vigorous-intensity physical activity (MVPA) within the 2-h postprandial window, daily LPA and MVPA during the 24 h before the meal, and sleep duration the night before [9–11]. GGIR performed an auto-calibration using local gravity and detected non-wear time based on the standard deviation and the range of raw acceleration signals in three axes. Technical details for autocalibration and non-wear detection were published by the GGIR developers online elsewhere [10–13]. Raw accelerometer data were aggregated into epochs of 5 s in ‘Euclidean Norm Minus One’ (ENMO), which were extracted from the GGIR output metadata to identify LPA and MVPA epochs using ENMO thresholds of 40 milligravity for LPA and 100 milligravity for MVPA [11, 14–16]. Subsequently, we computed total physical activity durations regardless of bout length, consistent with the updated 2020 World Health Organization physical activity guidelines [17]. The total LPA duration was defined as the sum of LPA epochs (number of LPA epochs * 5 s) in the respective time window (2-h postprandial window or 24-h window before the meal). The same calculation was applied to MVPA. Of note, the mean proportion of accelerometer wear time exceeded 99% of the 24 h on all the days included in our final analysis, with each day having over 16 h of wear time. This high accelerometer wearing compliance was consistent with this study’s strict inclusion criteria for meals mentioned in the subsequent section. More details are in Supplementary **Methods**.

### Glucose measures via CGM

Participants’ interstitial glucose was measured every 15 min throughout the nine days via a CGM device (Freestyle Libre Pro iQ; Abbott Diabetes Care) fitted on the upper, non-dominant arm. The CGM data were masked to the participants. The data were used to compute the study outcome postprandial glucose response and the pre-prandial glucose level for each meal. The postprandial glucose response was calculated using the 2-h incremental area under the curve (iAUC) of postprandial glucose levels based on the trapezoidal rule [18]. Mean glucose level within the 2-h window before each meal was computed to reflect the pre-prandial glucose level.

### Statistical analysis

A meal event was included in the analysis if all of the following criteria were met: (1) the presence of time-matched CGM measures, (2) no food intake within 2.5 h prior to the meal, and (3) consumed between 6:00 and 24:00 h (98.5% meals). We excluded participants with less than three qualifying meals from the analysis.

For the included participants and meals, baseline characteristics and longitudinal free-living measures were summarized using mean with standard deviation for continuous variables and count with percentage for categorical variables. The summary of longitudinal measures was based on each person’s average level over the free-living period.

A longitudinal repeated measure collected from multiple participants naturally contains two components: a between-person component reflecting the stable characteristic difference varying between individuals and a within-person component reflecting the situational fluctuations within individuals. We used the person-mean centering approach to separate the two components for each longitudinal free-living diet, physical activity, and sleep measure to derive the corresponding within-person and between-person variables [6] (Supplementary **Methods**). We also assessed the proportion of total variance attributable to the between-person variability component for each longitudinal measure using intraclass correlation coefficients (ICC). The proportion of total variance attributable to the within-person variability component equals 1 – ICC. Variance decomposition for ICC estimation was conducted using linear mixed-effect models (for continuous measures) or generalized linear mixed-effect models (for binary measures) with person-specific random intercepts.

Linear mixed-effect models with person-specific random intercepts were used to estimate the associations between the exposures and the outcome postprandial glucose iAUC. The exposures included the derived between-person and within-person lifestyle variables (i.e., diet, physical activity, and sleep measures) and meal time. The covariates included baseline characteristics (i.e., age, sex, ethnicity, education level, cigarette smoking, alcohol consumption, and body mass index) and mean 2-h pre-prandial glucose level. For each exposure, the association with the outcome was assessed in two models: a basic model adjusted for the covariates and a full model mutually adjusted for all other exposures in addition to the covariates. Bootstrapping was used to estimate 95% confidence intervals of regression coefficients. A positive between-person regression coefficient reflects that, on average, people with higher habitual levels of the lifestyle variable tended to have higher postprandial glucose iAUC. In contrast, a positive within-person regression coefficient reflects that, on average, a person tended to have higher postprandial glucose iAUC at meals when their lifestyle variable was higher than their usual level. An advantage of the analysis of within-person associations is that individuals act as their own control, removing influence from between-person variation that may result in imprecision and confounding. Of note, given the compositional nature of the movement behaviors (sleep, LPA, MVPA, and inactivity) and the near full accelerometer wear time for the data used, the regression estimates for sleep, LPA, and MVPA in our full model implicitly accounted for the isotemporal substitution of inactivity with these behaviors.

Because of known differences in metabolic regulation between sexes and individuals with different glycemic statuses, we conducted sensitivity analyses to check whether our main results were consistent in these subgroups [19]. We tested for interaction by including multiplicative interaction terms of sex or prediabetes status with exposure variables in the multivariable models. We also conducted stratified analyses by sex and prediabetes status. Because we only had two potential effect modifiers of interest and the stratified analyses are of general clinical relevance, we computed all stratified results instead of using models with interaction terms to pre-screen the potential effect modifiers. Because models with interaction terms had more independent variable terms and participant subgroups had smaller sample sizes, we excluded the between-person lifestyle exposures from models in the sensitivity analyses to limit loss in statistical power.

All analyses were conducted using R software (version 4.3.1). R packages ‘lme4’ and ‘lmerTest’ were used for linear mixed-effect models. Hypothesis tests were 2-sided. *P*-values less than 0.05 were considered statistically significant. *P*-values for interaction between 0.05 and 0.10 were considered marginally statistically significant.

## Results

### Baseline characteristics and longitudinal measures

The meal analysis included 789 participants with a mean age of 41 (SD 14) years (Table 1). Of these participants, 36% were males, 75% were ethnic Chinese, and 25% were ethnic Malay or Indian. During the study period, participants responded to 92% of EMA surveys and reported 13,424 meals, 11,333 (84%) of which met the inclusion criteria for the analyses (Supplementary eTable 1, Table 1). Refined grains were the most consumed meal food (68% of meals), followed by vegetables (42%), chicken (25%), red meat (24%), eggs (22%), seafood (21%), whole grains (17%), fruits (13%), soy food (10%), deep-fried food (10%), beans or nuts (8%), dairy (7%), and sweet desserts (7%). The mean postprandial glucose iAUC was 137.4 (SD 71.0) mmol/l*minute. The mean duration of postprandial physical activity was 0.3 (SD 0.1) hours for LPA and 0.2 (SD 0.1) hours for MVPA. Furthermore, the mean daily physical activity duration was 2.8 (SD 0.8) hours for LPA and 1.6 (SD 0.6) hours for MVPA. The mean sleep duration was 5.5 (SD 1.0) hours.

**Table 1:** Participant characteristics and longitudinal free-living measures

Characteristics	All participants (*N* = 789)
Age (years)	41 ± 14
Sex: Male	286 (36%)
Ethnicity: Chinese	589 (75%)
Malay	127 (16%)
Indian	73 (9%)
Education: Secondary and below	92 (12%)
A-level	229 (29%)
University and above	468 (59%)
Current cigarette smoking	95 (12%)
Heavy alcohol consumption	64 (8%)
BMI (kg/m^2^)	24 ± 5
Fasting plasma glucose (mmol/l)	5.0 ± 0.5
HbA1c (%)	5.4 ± 0.3
Mean glucose by CGM (mmol/l)	5.6 ± 0.6
Coefficient of variation by CGM (%)	19.2 ± 4.7
Prediabetes: Yes	204 (26%)
**Longitudinal free-living measures**	
Number of meals with CGM data (% total meals)	12,485 (93%)
Number of meals included for analysis (% meals with CGM data)	11,333 (91%)
% Meal time: 06:00–12:00 h	29 ± 17
12:00–18:00 h	36 ± 15
18:00–24:00 h	35 ± 15
Postmeal satiety (lowest 1 to highest 6)	3.8 ± 0.7
Pre-prandial mean blood glucose (mmol/l)	5.4 ± 0.6
Postprandial glucose iAUC (mmol/l*minute)	137.4 ± 71.0
Postprandial light-intensity physical activity (h)	0.3 ± 0.1
Postprandial moderate-to-vigorous-intensity physical activity (h)	0.2 ± 0.1
Daily light-intensity physical activity (h)	2.8 ± 0.8
Daily moderate-to-vigorous-intensity physical activity (h)	1.6 ± 0.6
Sleep duration (h)	5.5 ± 1.0

Data were in mean ± standard deviation or N (%). The definition for prediabetes was having fasting plasma glucose > = 5.6 mmol/l or HbA1c > = 5.7% measured during baseline physical examination. Criteria for meals to be included for analysis: (1) with time-matched CGM data, (2) consumed during 06:00–24:00 h of the day, and (3) without food consumption within 2.5 h before the meal. Postprandial measures were for the 2-h window after eating. Daily LPA and daily MVPA were measured during the 24 h before the meal. CGM: continuous glucose monitoring; iAUC: incremental area under the curve

### Within-person and between-person variation in longitudinal measures

**Fig. 2:**
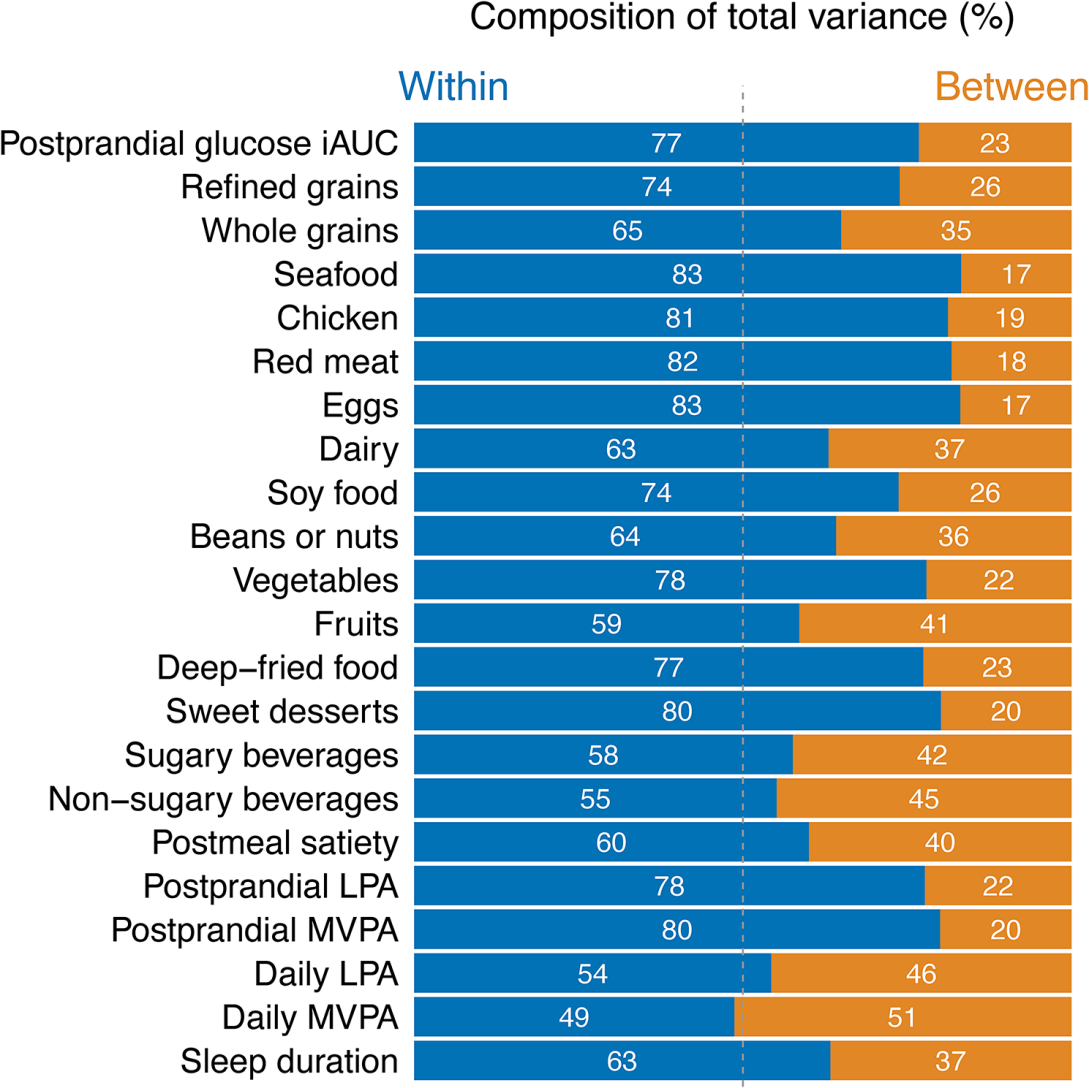
Relative contribution of within-person (in blue) and between-person (in orange) variation in longitudinal free-living measures. Postprandial measures were for the 2-h window after eating. Daily LPA and daily MVPA were measured during the 24 h before the meal. iAUC: incremental area under the curve; LPA: light-intensity physical activity; MVPA: moderate-to-vigorous-intensity physical activity

Figure 2 illustrates the relative contribution of within-person and between-person variations in longitudinal measures related to meals over the study period. For postprandial glucose responses (iAUC), the proportion of variation due to within-person variation was 77%, indicating greater variation within than between persons. Furthermore, all dietary and lifestyle factors varied more within than between individuals, except for daily MVPA.

### Association of lifestyle and contextual measures with postprandial glucose

We observed stronger associations at the within-person than the between-person level. At the within-person level (Table 2), participants had higher postprandial glucose responses (iAUC in mmol/l*min) when their meals contained refined grains (β: 46.2; 95% CI: 40.3, 52.1), whole grains (β: 23.8; 95% CI: 16.6, 31.0), and deep-fried food (β: 11.6; 95% CI: 4.3, 19.0) and when they had higher-than-usual post-meal satiety (β: 7.7; 95% CI: 5.1, 10.4) in the fully adjusted model. In comparison, participants had lower postprandial glucose iAUC when they consumed seafood (β: -8.6; 95% CI: -13.9, -3.3), eggs (β: -5.2; 95% CI: -10.2, -0.2), dairy (β: -13.5; 95% CI: -21.4, -5.6), beans or nuts (β: -9.2; 95% CI: -17.0, -1.4), fruits (β: -7.1; 95% CI: -13.5, -0.6), and non-sugary beverages (β: -9.5; 95% CI: -16.3, -2.8). Directions of associations changed from the basic to the fully adjusted model for whole grains. Correlation analysis suggested that this may be due to confounding by refined grains consumption that had a strong inverse correlation with whole grains consumption (Supplementary eFigure 1). Participants had lower postprandial glucose iAUC when they engaged in longer postprandial MVPA (β: -58.0; 95% CI: -73.8, -42.3), postprandial LPA (β: -24.7; 95% -39.5, -9.9), daily LPA during 24 h before the meal (β: -7.5; 95% CI: -10.7, -4.2), and sleep the night before (β: -2.7; 95% CI: -4.4, -1.0). At the between-person level, higher postprandial glucose responses were found in participants who habitually consumed more refined grains (β: 51.7; 95% CI: 22.5, 80.1) (Supplementary eTable 2). Regarding time of the day, postprandial glucose responses were the lowest for meals consumed in the morning (06:00–12:00 h) and the highest for meals consumed in the afternoon (12:00–18:00 h) (Table 2).

**Table 2:** Associations of within-person lifestyle exposures and eating time with postprandial glucose iAUC at 11,333 meals from 789 participants

	Basic model		Full model	
	Estimate (95% CI)	*p*-value	Estimate (95% CI)	*p*-value
**Within-person exposures**				
Meal composition				
Refined grains	42.5 (37.6, 47.3)	**< 0.001**	46.2 (40.3, 52.1)	**< 0.001**
Whole grains	-18.7 (-24.8, -12.7)	**< 0.001**	23.8 (16.6, 31.0)	**< 0.001**
Seafood	6.3 (1.0, 11.6)	**0.020**	-8.6 (-13.9, -3.3)	**0.002**
Chicken	12.6 (7.4, 17.7)	**< 0.001**	-2.6 (-7.8, 2.6)	0.330
Red meat	12.5 (7.4, 17.6)	**< 0.001**	-4.2 (-9.4, 1.0)	0.116
Eggs	-4.1 (-9.3, 1.1)	0.126	-5.2 (-10.2, -0.2)	**0.042**
Dairy	-29.4 (-37.5, -21.3)	**< 0.001**	-13.5 (-21.4, -5.6)	**0.001**
Soy food	6.4 (-0.9, 13.6)	0.084	-2.2 (-9.3, 4.8)	0.530
Beans or nuts	-9.5 (-17.7, -1.3)	**0.023**	-9.2 (-17.0, -1.4)	**0.021**
Vegetables	20.0 (15.4, 24.5)	**< 0.001**	0.8 (-4.0, 5.6)	0.742
Fruits	-11.0 (-17.7, -4.3)	**0.001**	-7.1 (-13.5, -0.6)	**0.031**
Deep-fried food	9.3 (1.8, 16.8)	**0.015**	11.6 (4.3, 19.0)	**0.002**
Sweet desserts	-2.5 (-10.8, 5.9)	0.558	4.0 (-4.0, 12.0)	0.331
Sugary beverages	-5.6 (-12.1, 1.1)	0.099	-1.2 (-7.6, 5.2)	0.716
Non-sugary beverages	-21.5 (-28.4, -14.6)	**< 0.001**	-9.5 (-16.3, -2.8)	**0.006**
Postmeal satiety	15.6 (13.0, 18.1)	**< 0.001**	7.7 (5.1, 10.4)	**< 0.001**
Postprandial light-intensity physical activity (h)	-62.6 (-75.9, -49.2)	**< 0.001**	-24.7 (-39.5, -9.9)	**0.001**
Postprandial moderate-to-vigorous-intensity physical activity (h)	-87.8 (-102.0, -73.6)	**< 0.001**	-58.0 (-73.8, -42.3)	**< 0.001**
Daily light-intensity physical activity (h)	-4.1 (-6.7, -1.6)	**0.002**	-7.5 (-10.7, -4.2)	**< 0.001**
Daily moderate-to-vigorous-intensity physical activity (h)	-1.8 (-5.3, 1.6)	0.304	1.8 (-2.5, 6.1)	0.411
Sleep duration (h)	-1.8 (-3.6, 0.0)	0.055	-2.7 (-4.4, -1.0)	**0.002**
**Meal time of the day**				
06:00–12:00 h	Reference			
12:00–18:00 h	62.5 (57.4, 67.6)	**< 0.001**	54.4 (48.8, 59.9)	**< 0.001**
18:00–24:00 h	49.3 (44.1, 54.5)	**< 0.001**	42.9 (36.9, 48.6)	**< 0.001**

Basic models were adjusted for age, sex, ethnicity, education, smoking, alcohol, body mass index, and mean 2-h pre-prandial glucose level as covariates. The full model was additionally mutually adjusted for eating time and all within-person and between-person exposures of diet, physical activity, and sleep. The unit of outcome postprandial glucose iAUC is mmol/l*minute. Postprandial measures were taken during the 2-h window after eating. Daily light-intensity physical activity and daily moderate-to-vigorous-intensity physical activity were measured during the 24 h before the meal. iAUC: incremental area under the curve

**Fig. 3:**
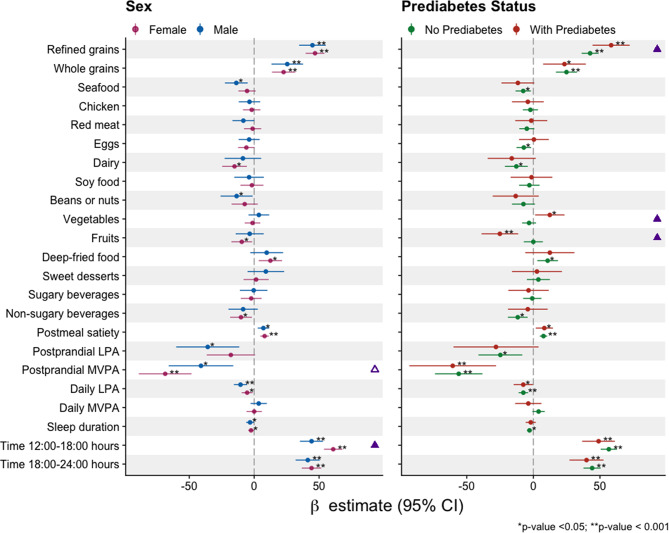
Associations of lifestyle exposures and meal time with postprandial glucose iAUC, stratified by sex and prediabetes status subgroups. The models were adjusted for age, sex, ethnicity, education level, smoking, alcohol, body mass index, and mean 2-h pre-prandial glucose level, except when the covariate was used to stratify. The models were also mutually adjusted for meal time, diet, physical activity, and sleep measures. The ▲ symbol indicates a significant interaction term (*p*-value < 0.05), and the △ symbol indicates a marginally significant interaction term (0.05 < = *p*-value < 0.1) (for details, see Supplementary eTable 3). Postprandial measures were for the 2-h window after eating. Daily LPA and daily MVPA were measured during the 24 h before the meal. iAUC: incremental area under the curve. LPA: light-intensity physical activity; MVPA: moderate-to-vigorous-intensity physical activity

Consistent results were found in the stratified analysis by sex and prediabetes status, with a few exceptions (Fig. 3). Compared with females, males had a smaller increase in postprandial glucose responses in the afternoon versus the morning. The association between postprandial MVPA and lower postprandial glucose responses tended to be weaker in males than in females (*p*-value = 0.096). Participants with prediabetes had a larger increase in postprandial glucose responses when consuming refined grains and vegetables and a larger decrease in postprandial glucose responses when consuming fruits in meals.

## Discussion

This intensive longitudinal observational study assessed associations of diet, physical activity, and sleep with postprandial glucose levels under free-living conditions for meals in Asian adults without diabetes. Higher consumption of grains, particularly refined grains, and deep-fried food and lower consumption of protein-rich foods as part of meals were associated with higher postprandial glucose levels. Longer postprandial physical activity, especially postprandial MVPA, was associated with substantially lower postprandial glucose levels. Longer daily LPA and sleep duration during the previous night were also associated with lower postprandial glucose levels. Finally, postprandial glucose levels were lower in the morning than later in the day.

Traditional epidemiological studies have focused primarily on between-person differences in habitual levels of lifestyle behaviors. A growing appreciation of temporal within-person variations of lifestyle behaviors has been fostered by recent studies facilitated by technological advancements in data collection [20, 21]. Leveraging intensive longitudinal measures, we evaluated variances and associations at both between-person and within-person levels. Notably, most of the examined lifestyle and glucose measures varied relatively more within persons than between persons, and associations of the lifestyle factors with postprandial glucose levels were stronger at the within-person level. The results of the within-person analysis indicate that lifestyle behaviors that individuals already engaged in some of the time in real-world settings were associated with substantially lower postprandial glucose levels. Hence, for many individuals, more consistent engagement in favorable lifestyle behaviors may be beneficial for postprandial glucose control even without the adoption of novel lifestyle behaviors.

Diet is a major determinant of the appearance and disposal of circulating glucose in the postprandial state [1, 5]. Informed mostly by studies with small samples or controlled meals, recent reviews have identified several dietary strategies to attenuate postprandial glucose responses for individuals without diabetes, including reducing total carbohydrate intake, selecting carbohydrates with lower glycemic index, adding fiber, and increasing proportions of healthy protein and fat [1, 5]. Consistent with these recommendations, we found significantly higher glucose levels following the consumption of carbohydrate-rich food. Particularly, the magnitude of glucose increment associated with refined grains consumption was about double the increment associated with whole grains consumption. Given that refined grains were consumed much more frequently than whole grains in our Asian study population, population interventions encouraging switching from refined grains to whole grains may improve glucose control substantially. Protein and fat intake can blunt postprandial glucose responses by slowing carbohydrate digestion and influencing glucose disposal [1, 5, 22, 23]. In our study, intake of protein-rich foods such as seafood and dairy was linked to substantially lower postprandial glucose levels. Consumption of nuts or beans and fruits was also associated with lower postprandial glucose levels. In contrast, intake of deep-fried food was linked to higher postprandial glucose levels. This may partly reflect the popularity of high-carbohydrate deep-fried food in Singapore. Furthermore, fried food consumption was associated with a higher risk of type 2 diabetes in large US prospective cohorts [24, 25].

Our results were largely consistent across subgroups by sex and prediabetes status. However, having prediabetes amplified the increase in postprandial glucose levels when consuming refined grains and the decrease when consuming fruits. This result suggests that persons with prediabetes may benefit more from consuming less refined grains or more fruits for postprandial glucose control than normoglycemic individuals. Unlike participants without prediabetes, participants with prediabetes had higher postprandial glucose levels when consuming vegetables. This unexpected observation may be driven by the consumption of starchy vegetables, but requires further study. Our interaction analyses had lower statistical power, so these results should be interpreted with caution and need to be confirmed in future studies.

Chrono-nutrition studies have consistently found higher postprandial glucose responses to standardized isocaloric meals in the evening and at midnight than in the morning [1, 5]. Our results based on free-choice meals are consistent with these earlier results. Nevertheless, few previous studies investigated postprandial glucose responses to meals consumed in the afternoon [26]. Our study addressed this gap and showed that postprandial glucose responses did not increase monotonously from the morning to the evening but peaked in the afternoon. This observation persisted in our full model, controlling for key lifestyle measures available, including meal food groups, physical activity, and post-meal satiety. Several other factors may contribute to our observation, including circadian rhythms of glucose-regulating hormones and insulin resistance and fluctuations in psychological stress throughout the day [27, 28]. For example, insulin resistance and psychological stress have been linked to higher blood glucose levels [27, 28]. While insulin resistance tends to increase from morning to evening, psychological stress might peak in the afternoon in adults and result in higher glucose levels in the afternoon [27, 29]. It is also unclear whether our findings are specific to eating patterns in our Asian population. More research on chronological variation in detailed food choices and glucose metabolism in diverse populations is needed to better understand changes in postprandial glucose responses during the day.

Physical activity improves insulin sensitivity and insulin-independent muscle glucose uptake [1]. In our study, longer physical activity, particularly postprandial MVPA, was associated with substantially lower glucose levels after meals. Of note, our study population had an average of about 10 min MVPA during the 2-h postprandial time window, 97% of which was of moderate intensity, and only 3% was of vigorous intensity (with ENMO > = 400 milligravity). Postprandial vigorous-intensity physical activity may be more effective than light- or moderate-intensity physical activity in reducing postprandial glucose levels but is often less feasible under real-world conditions [30, 31]. Our results suggest that promoting postprandial physical activity, especially moderate intensity activities such as brisk walking after eating, may considerably improve glucose control. Our findings are consistent with previous experimental studies investigating activity timing in adults with and without diabetes [1, 31–36]. For example, in the randomized crossover trials by DiPietro et al. and Pahra et al., three 15-minute bouts of postmeal walking were more effective in lowering postprandial glucose levels than 45 min of sustained morning or afternoon walking [35, 37]. In addition to the associations for postprandial physical activity, we found that engaging in daily LPA during the 24 h before the meal was modestly linked to lower postprandial glucose levels. In contrast, daily MVPA showed no such associations. This contradicts our hypothesis that daily MVPA would have a stronger inverse association than daily LPA. However, this observation aligns with the findings of a randomized crossover trial showing that LPA, but not MVPA, reduced glucose in persons with diabetes over the subsequent 24 h [38]. Further research is needed to elucidate how the interplay between the intensity and timing of physical activity affects glucose control. Overall, our findings support the glycemic benefits of physical activity and indicate that benefits for postprandial glucose control may be substantially enhanced by timing the activity after meals.

The link between sleep and glucose metabolism is well established [39–41]. However, few studies have been conducted on the relationship between sleep and postprandial glucose responses in free-living adults without diabetes. Tsereteli et al. recently found that short sleep duration interacted with meal type, observing a stronger association between short sleep duration and higher postprandial glucose responses to pure glucose than high-carbohydrate and high-fat breakfasts in 953 free-living healthy adults [40]. However, in contrast to our study they did not find a significant marginal association between sleep duration and postprandial glucose responses. The two studies differed in participant characteristics and study designs. For example, standardized meals were provided in Tsereteli et al.’s study, and the participants had a longer average sleep duration of 7.7 h (vs. 5.5 h in our study) [40]. Nevertheless, both results agree with evidence from laboratory and epidemiological studies that longer sleep durations generally improve postprandial glucose control [39–41].

This study has several strengths. First, we collected lifestyle and glycemic data continuously in real-time using mobile technologies. Instead of traditional methods relying on recalls, smartphone-based EMA surveys were used to collect data on recent food consumption. Glucose levels, physical activity, and sleep were objectively and frequently measured using CGM and accelerometers. These intensive longitudinal data enable analysis at the resolution of meal events and at both between-person and within-person levels. Second, our study had good compliance and response rates to continuous free-living measures. Participants responded to over 90% of EMA prompts. Furthermore, previous studies on lifestyle factors and postprandial glucose responses in free-living individuals were mostly conducted in Western populations. Our study provides insights into Asian populations.

Our study also had several limitations. To reduce participant burden and maintain high response rates, we did not include detailed dietary assessments. As a result, we were not able to estimate portion sizes or calculate nutrient intakes. Future studies with more detailed dietary assessments can provide further insights, especially with technologies allowing detailed dietary data collection with limited participant burden. Secondly, the timing of meals was self-reported, potentially leading to non-differential misclassification and reducing the strength of associations. Thirdly, our study assessed movement behaviors using data from wrist-worn accelerometers and did not cover the full complexity of these behaviors. The total duration of LPA and MVPA were derived based on activity intensity thresholds, which could introduce some misclassification. Moreover, the wrist-worn accelerometer data lacked the ability to accurately assess sedentary behavior due to the absence of posture information, so we did not specifically analyze sedentary behavior [42]. Future studies dedicated to a broader range of movement measures are warranted, especially those that leverage advanced methodologies such as machine learning algorithms and combined sensing (e.g., wrist- and thigh-worn accelerometers) for more accurate classification of movement behaviors. Fourthly, our study was observational, so we cannot rule out the possibility of residual confounding. Nevertheless, the extensive longitudinal measures enabled evaluations of within-person associations unaffected by confounding due to between-person differences. Fifthly, our participants were of working age without major chronic diseases. They had higher education levels, with 57% holding at least a university degree, compared to 37% in the general adult population of Singapore [43]. Therefore, their lifestyle behaviors may not fully represent those of the broader Singapore population. However, the lifestyle measures of our participants provided sufficient variability, which was more critical for our association study. In addition, given the difference across populations (e.g., distinct diet patterns compared to non-Asian countries), further research on other populations across the world is needed to evaluate the generalizability of our findings [44, 45]. Furthermore, although our study focuses on postprandial glucose levels, influences on non-glycemic aspects of health should also be considered in lifestyle choices.

## Conclusions

In summary, lower consumption of carbohydrate-rich foods (particularly refined grains) and deep-fried foods and higher consumption of protein-rich foods were associated with lower postprandial glucose levels in real-life settings. Longer durations of physical activity (particularly during the postprandial period) and sleep were also associated with lower postprandial glucose levels. Our results support multi-component lifestyle modifications focusing on diet, physical activity, and sleep behaviors to improve postprandial glucose control in adults without diabetes. Taking into account the timing of eating and physical activity may enhance the impact of these interventions.

## Electronic supplementary material

Below is the link to the electronic supplementary material.

Supplementary Material 1

Supplementary Material 2

## Data Availability

The data used for this study are held by the Saw Swee Hock School of Public Health at the National University of Singapore. The data may be released to bona fide researchers upon reasonable requests and agreements via https://blog.nus.edu.sg/sphs/data-and-samples-request/. The data release must conform to the Personal Data Protection Act in Singapore.

## Abbreviations

CGM: Continuous glucose monitor
EMA: Ecological momentary assessment
iAUC: Incremental area under the curve
LPA: Light-intensity physical activity
MVPA: Moderate-to-vigorous-intensity physical activity
