# Supplementary material for: Diet, physical activity, and sleep in relation to postprandial glucose responses under free-living conditions: an intensive longitudinal observational study
Source: Int J Behav Nutr Phys Act. 2024 Dec 18;21:142. doi: 10.1186/s12966-024-01693-5 (PMC11658231; doi:10.1186/s12966-024-01693-5)
Supplement: Supplementary file 1 — Supplementary Material 1 [file 12966_2024_1693_MOESM1_ESM.docx]

STROBE Statement—checklist of items that should be included in reports of observational studies

|  | Item No | Recommendation | Page  No |
| --- | --- | --- | --- |
| **Title and abstract** | 1 | (*a*) Indicate the study’s design with a commonly used term in the title or the abstract  - Observational study with intensive longitudinal data as stated in the Abstract | 2 |
|  |  | (*b*) Provide in the abstract an informative and balanced summary of what was done and what was found  - Provided in the Abstract | 2 |
| Introduction | | | |
| Background/rationale | 2 | Explain the scientific background and rationale for the investigation being reported  - Included in the Background introduction | 3 |
| Objectives | 3 | State specific objectives, including any prespecified hypotheses  - Included in the Background introduction | 3 |
| Methods | | | |
| Study design | 4 | Present key elements of study design early in the paper  - Included in the Methods | 4 |
| Setting | 5 | Describe the setting, locations, and relevant dates, including periods of recruitment, exposure, follow-up, and data collection  - Included in the Methods | 4 |
| Participants | 6 | (*a*) *Cohort study*—Give the eligibility criteria, and the sources and methods of selection of participants. Describe methods of follow-up  *Case-control study*—Give the eligibility criteria, and the sources and methods of case ascertainment and control selection. Give the rationale for the choice of cases and controls  *Cross-sectional study*—Give the eligibility criteria, and the sources and methods of selection of participants  - Included in the Methods | 4 |
|  |  | (*b*) *Cohort study*—For matched studies, give matching criteria and number of exposed and unexposed  *Case-control study*—For matched studies, give matching criteria and the number of controls per case  *-* Not applicable | Not applicable |
| Variables | 7 | Clearly define all outcomes, exposures, predictors, potential confounders, and effect modifiers. Give diagnostic criteria, if applicable  - Included in the Methods | 5-7 |
| Data sources/ measurement | 8* | For each variable of interest, give sources of data and details of methods of assessment (measurement). Describe comparability of assessment methods if there is more than one group  - Provided in the Methods | 4-6 |
| Bias | 9 | Describe any efforts to address potential sources of bias  - Provided in the Methods | 6-7 |
| Study size | 10 | Explain how the study size was arrived at  - Provided in the Methods and Results | 6-8 |
| Quantitative variables | 11 | Explain how quantitative variables were handled in the analyses. If applicable, describe which groupings were chosen and why  - Provided in the Methods | 6-8 |
| Statistical methods | 12 | (*a*) Describe all statistical methods, including those used to control for confounding  - Provided in the Methods | 6-8 |
|  |  | (*b*) Describe any methods used to examine subgroups and interactions  - Provided in the Methods | 7-8 |
|  |  | (*c*) Explain how missing data were addressed  - Provided in the Supplementary materials | s2 |
|  |  | (*d*) *Cohort study*—If applicable, explain how loss to follow-up was addressed  *Case-control study*—If applicable, explain how matching of cases and controls was addressed  *Cross-sectional study*—If applicable, describe analytical methods taking account of sampling strategy  *-* Not applicable | Not applicable |
|  |  | (*e*) Describe any sensitivity analyses  - Provided in the Methods by subgroup and interaction analysis | 6-8 |

Continued on next page

| Results | | | |
| --- | --- | --- | --- |
| Participants | 13* | (a) Report numbers of individuals at each stage of study—eg numbers potentially eligible, examined for eligibility, confirmed eligible, included in the study, completing follow-up, and analysed  - Provided in the Results. | 8 |
|  |  | (b) Give reasons for non-participation at each stage  *-* Not applicable | Not applicable |
|  |  | (c) Consider use of a flow diagram  - Not required. We provided the detail counts in the supplementary materials. | Not applicable |
| Descriptive data | 14* | (a) Give characteristics of study participants (eg demographic, clinical, social) and information on exposures and potential confounders  - Provided in the Results | 8 |
|  |  | (b) Indicate number of participants with missing data for each variable of interest  - Provided in the Supplementary materials | s2 |
|  |  | (c) *Cohort study*—Summarise follow-up time (eg, average and total amount)  *-* Not applicable | Not applicable |
| Outcome data | 15* | *Cohort study*—Report numbers of outcome events or summary measures over time  *-* Not applicable | Not applicable |
|  |  | *Case-control study—*Report numbers in each exposure category, or summary measures of exposure  *-* Not applicable | Not applicable |
|  |  | *Cross-sectional study—*Report numbers of outcome events or summary measures  *-* Not applicable | Not applicable |
| Main results | 16 | (*a*) Give unadjusted estimates and, if applicable, confounder-adjusted estimates and their precision (eg, 95% confidence interval). Make clear which confounders were adjusted for and why they were included  - Provided in the Results Table 2 | 20 |
|  |  | (*b*) Report category boundaries when continuous variables were categorized  - Provided in the Results Table 1 | 19 |
|  |  | (*c*) If relevant, consider translating estimates of relative risk into absolute risk for a meaningful time period  *-* Not applicable | Not applicable |
| Other analyses | 17 | Report other analyses done—eg analyses of subgroups and interactions, and sensitivity analyses  - Provided in the Results | 9 |
| Discussion | | | |
| Key results | 18 | Summarise key results with reference to study objectives  - Provided in the Discussion | 9-10 |
| Limitations | 19 | Discuss limitations of the study, taking into account sources of potential bias or imprecision. Discuss both direction and magnitude of any potential bias  - Provided in the Discussion | 13-14 |
| Interpretation | 20 | Give a cautious overall interpretation of results considering objectives, limitations, multiplicity of analyses, results from similar studies, and other relevant evidence  - Provided in the Discussion | 10-14 |
| Generalisability | 21 | Discuss the generalisability (external validity) of the study results  - Provided in the Discussion | 10-14 |
| Other information | | | |
| Funding | 22 | Give the source of funding and the role of the funders for the present study and, if applicable, for the original study on which the present article is based  - Provided after the Discussion | 15 |

*Give information separately for cases and controls in case-control studies and, if applicable, for exposed and unexposed groups in cohort and cross-sectional studies.

**Note:** An Explanation and Elaboration article discusses each checklist item and gives methodological background and published examples of transparent reporting. The STROBE checklist is best used in conjunction with this article (freely available on the Web sites of PLoS Medicine at http://www.plosmedicine.org/, Annals of Internal Medicine at http://www.annals.org/, and Epidemiology at http://www.epidem.com/). Information on the STROBE Initiative is available at www.strobe-statement.org.
