# Supplementary material for: Diet, physical activity, and sleep in relation to postprandial glucose responses under free-living conditions: an intensive longitudinal observational study
Source: Int J Behav Nutr Phys Act. 2024 Dec 18;21:142. doi: 10.1186/s12966-024-01693-5 (PMC11658231; doi:10.1186/s12966-024-01693-5)
Supplement: Supplementary file 2 — Supplementary Material 2 [file 12966_2024_1693_MOESM2_ESM.docx]

# **SUPPLEMENTARY MATERIALS (Methods and Results)**

# **Title**: Diet, physical activity, and sleep in relation to postprandial glucose responses under free-living conditions: an intensive longitudinal observational study

# **1. Supplementary Methods**

**Study participant**

Among the 859 participants recruited between May 2021 and April 2024 and provided time-matched CGM data, 39 were found to have diabetes based on data collected via questionnaire interview and physical examination at the baseline visit. Specifically, participants had diabetes if they met one or more of the following criteria: (1) reporting yes to ‘Has a doctor ever told you that you have diabetes (not during pregnancy)?’, (2) having fasting plasma glucose >= 7 mmol/l, and (3) having HbA1c >= 6.5%.

**Pre-defined Singapore food group lists for food logging**

Participants’ food intakes were assessed using check-all-that-apply questions (‘What foods were part of your meal/snack/drink?’) and three pre-defined Singapore food group lists. The meal food group list included refined grains (e.g., white rice, noodles, pasta, bread, or cereal), whole grains (e.g., brown or wholegrain rice, noodles, pasta, bread, or cereal), seafood, chicken, red meat (e.g., beef, pork, mutton, lamb), eggs, dairy products (e.g., milk, yogurt, cheese), soy food (e.g., tofu and tempeh), beans, peas, nuts, seeds (e.g., peanuts, dahl, sambar), vegetables, fruit, deep fried foods (e.g., puffs, samosas, French fries, fried chicken), and sweet desserts. The snack food group list included sweets (e.g., cookies, cakes, candy, sweet desserts), chips or fried crackers (e.g., potato chips, prawn crackers), other deep-fried food (e.g., puffs, samosas, French fries, fried chicken), nuts or seeds, and fruit or vegetables. The drink group list included various sugary and non-sugary beverages. Other food items that participants could not match directly to the pre-defined food group were recorded using free texts. These free-text food entries were independently examined by two researchers and were mapped to or merged with the closest food groups in the pre-defined lists, with disagreements resolved by consensus or by the decision of a third researcher.

**Processing of accelerometer data to derive physical activity and sleep measures**

Participants were instructed to wear accelerometer at all times during the nine study days. The accelerometer was fitted using Axivity Wrist Band and in line with the mounting convention stated in <https://axivity.com/userguides/ax3/using/>. Raw accelerometer data were processed using R package GGIR (version 3.1-1) into epochs of 5 seconds to derive durations of physical activity and sleep specific to each meal, including light-intensity physical activity (LPA) and moderate-to-vigorous-intensity physical activity (MVPA) within the 2-hour postprandial window, daily LPA and MVPA during the 24 hours before the eating event, and sleep duration the night before the eating. Total physical activity durations were used, including the bouted and non-bouted portions. Algorithms developed by van Hees et al. were used to pre-process the acceleration signals and to detect sustained inactivity and sleep [1–3]. Euclidean Norm Minus One was the acceleration metric to define LPA and MVPA, with the lower acceleration bound being 40 milligravity for LPA, 100 milligravity for MVPA, and 400 milligravity for vigorous physical activity, where 1 milligravity = 0.00981 m.s^−2^ [4–6]. Multivariate imputation by chained equations with 100 iterations was applied to impute small proportions of missing data (3% of physical activity measures and 5% for sleep duration) via R package mice (version 3.16.0).

**The person-mean centering approach**

We used the person-mean centering approach to derive the corresponding within-person and between-person variables for each longitudinal variable. Specifically, if we denote the value of a longitudinal variable Χ for person *i* at eating event *j* as $Xij$, the person-specific mean $\overline{X}i$ makes the between-person variable, while the deviation from the person-specific mean ($Xij-\overline{X}i$) makes the within-person variable. The derivation was performed for variables on diet, physical activity, and sleep behaviors. Notably, raw food group variables were binary; therefore, the derived between-person variable of a food group reflected the proportion of each person’s eating events that contained the food group.

**References**

1. van Hees VT, Gorzelniak L, Dean León EC, Eder M, Pias M, Taherian S, et al. Separating Movement and Gravity Components in an Acceleration Signal and Implications for the Assessment of Human Daily Physical Activity. PLoS One. 2013;8:e61691.

2. van Hees VT, Sabia S, Jones SE, Wood AR, Anderson KN, Kivimäki M, et al. Estimating sleep parameters using an accelerometer without sleep diary. Sci Rep. 2018;8:12975.

3. Migueles JH, Rowlands A V., Huber F, Sabia S, van Hees VT. GGIR: A Research Community–Driven Open Source R Package for Generating Physical Activity and Sleep Outcomes From Multi-Day Raw Accelerometer Data. J Meas Phys Behav. 2019;2:188–96.

4. Le Cornu Q, Chen M, van Hees V, Léger D, Fayosse A, Yerramalla MS, et al. Association of physical activity, sedentary behaviour, and daylight exposure with sleep in an ageing population: findings from the Whitehall accelerometer sub-study. International Journal of Behavioral Nutrition and Physical Activity. 2022;19:144.

5. Chen M, Landré B, Marques-Vidal P, van Hees VT, van Gennip ACE, Bloomberg M, et al. Identification of physical activity and sedentary behaviour dimensions that predict mortality risk in older adults: development of a machine learning model in the Whitehall II accelerometer sub-study and external validation in the CoLaus study. EClinicalMedicine. 2023;55:101773.

6. Sabag A, Ahmadi MN, Francois ME, Postnova S, Cistulli PA, Fontana L, et al. Timing of Moderate to Vigorous Physical Activity, Mortality, Cardiovascular Disease, and Microvascular Disease in Adults With Obesity. Diabetes Care. 2024;47:890–7.

# **2. Supplementary Results**

**eTable 1**: Adherence to longitudinal free-living measurements and meal composition

| **Characteristics** | **All participants (n = 789)** |
| --- | --- |
| Number of responded EMA (% sent) | 39275 (92%) |
| Minutes to start EMA since sent | 14.2 ± 12.8 |
| Minutes to complete EMA | 1.5 ± 2.4 |
| Number of meals from EMA (% responded) | 13424 (33%) |
| Number of meals with CGM (% meals) | 12485 (93%) |
| Number of days per person with meals and CGM | 7.9 ± 1.8 |
| Number of meals with CGM per person | 15.8 ± 6.1 |
| Number of meals with CGM per day per person | 2.0 ± 0.5 |
| Number of meals included for analysis (% meals with CGM) | 11333 (91%) |
| Number of days with meals included for analysis per person | 7.7 ± 1.8 |
| Number of meals included for analysis per person | 14.4 ± 5.6 |
| Number of meals included for analysis per day per person | 1.8 ± 0.5 |
| Meal composition (%) |  |
| Refined grains | 68 ± 24 |
| Whole grains | 17 ± 19 |
| Seafood | 21 ± 17 |
| Chicken | 25 ± 19 |
| Red meat | 24 ± 18 |
| Eggs | 22 ± 17 |
| Dairy | 7 ± 12 |
| Soy food | 10 ± 13 |
| Beans or nuts | 8 ± 13 |
| Vegetables | 42 ± 25 |
| Fruits | 13 ± 18 |
| Deep-fried food | 10 ± 13 |
| Sweet desserts | 7 ± 10 |
| Sugary beverages | 15 ± 19 |
| Non-sugary beverages | 13 ± 18 |

Data were in mean ± standard deviation or N (%). Criteria for meals to be included for analysis: (1) with time-matched CGM data, (2) consumed during 06:00-24:00 hours of the day, and (3) without food consumption within 2.5 hours before the meal event. The summary of longitudinal measures was based on each person’s average level over the free-living period. Pre-prandial and postprandial measures were based on the 2-hour window before and after meals. 97% of the postprandial moderate-to-vigorous-intensity physical activity was of moderate intensity for meals. EMA ecological momentary assessment; CGM: continuous glucose monitoring; iAUC: incremental area under the curve.

**eTable 2**: Associations of between-person lifestyle exposures with postprandial glucose iAUC at 11,333 meals from 789 participants

|  | **Basic model** | | **Full model** | |
| --- | --- | --- | --- | --- |
|  | **Estimate (95% CI)** | **p-value** | **Estimate (95% CI)** | **p-value** |
| **Between-person exposures** | | | | |
| Meal composition |  |  |  |  |
| Refined grains | 43.2 (21.0, 65.3) | **<0.001** | 51.7 (22.5, 81.0) | **0.001** |
| Whole grains | -12.2 (-40.7, 16.2) | 0.403 | 35.5 (-2.2, 73.3) | 0.071 |
| Seafood | -10.0 (-40.1, 20.0) | 0.516 | -16.9 (-49.4, 15.5) | 0.317 |
| Chicken | 11.5 (-16.8, 39.9) | 0.428 | 9.3 (-20.8, 39.5) | 0.553 |
| Red meat | -1.0 (-30.4, 28.3) | 0.945 | -14.9 (-47.2, 17.5) | 0.377 |
| Eggs | -22.8 (-52.3, 6.6) | 0.132 | -19.6 (-52.1, 12.8) | 0.245 |
| Dairy | -35.2 (-78.6, 8.2) | 0.115 | -17.3 (-65.0, 30.5) | 0.487 |
| Soy food | -4.2 (-43.7, 35.3) | 0.836 | 0.8 (-44.0, 45.9) | 0.973 |
| Beans or nuts | -25.5 (-66.2, 15.3) | 0.224 | -28.1 (-75.6, 19.3) | 0.255 |
| Vegetables | 6.7 (-14.9, 28.3) | 0.545 | 14.6 (-11.3, 40.4) | 0.277 |
| Fruits | -1.6 (-32.8, 29.5) | 0.919 | 23.7 (-10.9, 58.3) | 0.188 |
| Deep-fried food | -12.0 (-55.8, 32.0) | 0.596 | -21.6 (-68.0, 25.0) | 0.371 |
| Sweet desserts | -30.6 (-84.6, 23.3) | 0.269 | -33.7 (-90.0, 22.8) | 0.251 |
| Sugary beverages | 2.2 (-24.8, 29.2) | 0.874 | 6.8 (-21.6, 35.2) | 0.646 |
| Non-sugary beverages | -28.3 (-57.5, 1.0) | 0.060 | -19.3 (-50.4, 11.9) | 0.235 |
| Postmeal satiety | -24.3 (-81.7, 32.9) | 0.408 | 51.7 (-60.2, 163.3) | 0.374 |
| Postprandial light-intensity physical activity (hour) | -35.6 (-97.5, 26.3) | 0.263 | -7.9 (-122.8, 107.1) | 0.895 |
| Postprandial moderate-to-vigorous-intensity physical activity (hour) | -5.5 (-12.4, 1.5) | 0.125 | -9.3 (-23.3, 4.7) | 0.202 |
| Daily light-intensity physical activity (hour) | -4.4 (-12.5, 3.7) | 0.290 | 1.5 (-13.8, 16.7) | 0.853 |
| Daily moderate-to-vigorous-intensity physical activity (hour) | -3.7 (-9.4, 2.0) | 0.206 | -4.2 (-10.2, 1.8) | 0.178 |
| Sleep duration (hour) | 6.3 (-1.2, 13.8) | 0.101 | 5.1 (-2.6, 12.9) | 0.203 |

Basic models were adjusted for age, sex, ethnicity, education, smoking, alcohol, body mass index, and mean 2-hour pre-prandial glucose level as covariates. The full model was additionally mutually adjusted for eating time and all within-person and between-person exposures of diet, physical activity, and sleep. The unit of outcome postprandial glucose iAUC is mmol/l*minute. Postprandial measures were taken during the 2-hour window after eating. iAUC: incremental area under the curve.

**eTable 3:** Interaction effects for sex and pre-diabetes status with lifestyle exposures and meal time on postprandial glucose iAUC at 11333 meals from 789 participants

|  | **Model with interaction terms for sex** | | **Model with interaction terms for prediabetes status** | |
| --- | --- | --- | --- | --- |
|  | **Estimate (95% CI)** | **p-value** | **Estimate (95% CI)** | **p-value** |
| **Interaction terms** | | | | |
| Meal composition |  |  |  |  |
| Refined grains | -2.0 (-14.6, 10.5) | 0.750 | 15.7 (1.8, 29.6) | **0.027** |
| Whole grains | 2.9 (-12.3, 18.0) | 0.711 | -1.3 (-17.6, 15.0) | 0.878 |
| Seafood | -8.4 (-19.5, 2.6) | 0.137 | -4.2 (-16.6, 8.1) | 0.502 |
| Chicken | -1.7 (-12.4, 9.0) | 0.754 | -2.0 (-14.0, 10.0) | 0.742 |
| Red meat | -7.0 (-17.8, 3.7) | 0.199 | 3.5 (-8.6, 15.6) | 0.573 |
| Eggs | 2.2 (-8.1, 12.6) | 0.673 | 7.6 (-3.7, 19.0) | 0.188 |
| Dairy | 6.5 (-10.5, 23.6) | 0.452 | -3.4 (-21.6, 14.8) | 0.716 |
| Soy food | -2.3 (-16.7, 12.2) | 0.758 | 1.4 (-14.5, 17.3) | 0.862 |
| Beans or nuts | -6.2 (-22.2, 9.8) | 0.448 | -5.7 (-23.3, 11.9) | 0.529 |
| Vegetables | 4.9 (-5.1, 14.9) | 0.335 | 15.6 (4.5, 26.8) | **0.006** |
| Fruits | 6.2 (-7.3, 19.6) | 0.368 | -24.9 (-39.1, -10.7) | **0.001** |
| Deep-fried food | -3.0 (-18.6, 12.6) | 0.708 | 1.6 (-16.6, 19.7) | 0.867 |
| Sweet desserts | 7.6 (-9.5, 24.8) | 0.385 | -0.8 (-19.5, 18.0) | 0.936 |
| Sugary beverages | 1.8 (-11.5, 15.1) | 0.788 | -3.1 (-18.2, 12.1) | 0.693 |
| Non-sugary beverages | 1.6 (-12.5, 15.7) | 0.827 | 7.5 (-7.7, 22.7) | 0.334 |
| Postmeal satiety | -0.9 (-6.4, 4.6) | 0.750 | 0.7 (-5.7, 7.0) | 0.836 |
| Postprandial light-intensity physical activity (hours) | -17.7 (-48.4, 12.9) | 0.258 | -2.8 (-35.7, 30.0) | 0.866 |
| Postprandial moderate-to-vigorous-intensity physical activity (hours) | 27.4 (-4.7, 59.6) | 0.096 | -4.8 (-38.9, 29.3) | 0.784 |
| Daily light-intensity physical activity (hours) | -5.0 (-11.6, 1.6) | 0.138 | 0.0 (-7.3, 7.4) | 0.998 |
| Daily moderate-to-vigorous-intensity physical activity (hours) | 3.5 (-5.2, 12.1) | 0.431 | -7.8 (-17.7, 2.2) | 0.126 |
| Sleep duration (hours) | -0.7 (-4.4, 3.0) | 0.708 | 0.9 (-2.9, 4.8) | 0.636 |
| Meal time of the day (reference: 06:00-12:00 hours) | | | | |
| 12:00-18:00 hours | -18.2 (-29.4, -7.1) | **0.001** | -9.4 (-21.7, 2.8) | 0.132 |
| 18:00-24:00 hours | -4.6 (-16.2, 7.1) | 0.446 | -6.0 (-18.8, 6.9) | 0.363 |
| **Non-interaction terms (main effect terms)** | | | | |
| Meal composition |  |  |  |  |
| Refined grains | 46.8 (39.6, 54.1) | **<0.001** | 42.6 (35.8, 49.3) | **<0.001** |
| Whole grains | 22.6 (13.7, 31.5) | **<0.001** | 24.8 (16.4, 33.2) | **<0.001** |
| Seafood | -5.5 (-12.2, 1.2) | 0.109 | -7.6 (-13.7, -1.5) | **0.015** |
| Chicken | -1.9 (-8.6, 4.8) | 0.577 | -2.3 (-8.3, 3.8) | 0.461 |
| Red meat | -1.3 (-7.9, 5.4) | 0.708 | -5.0 (-11.0, 1.0) | 0.105 |
| Eggs | -6.0 (-12.3, 0.4) | **0.066** | -7.2 (-13.0, -1.4) | **0.016** |
| Dairy | -15.2 (-24.7, -5.7) | **0.002** | -12.9 (-22.0, -3.8) | **0.006** |
| Soy food | -1.7 (-10.6, 7.2) | 0.709 | -3.0 (-11.1, 5.2) | 0.476 |
| Beans or nuts | -7.4 (-17.5, 2.7) | 0.151 | -7.5 (-16.6, 1.7) | 0.109 |
| Vegetables | -1.3 (-7.3, 4.7) | 0.678 | -3.2 (-8.8, 2.3) | 0.258 |
| Fruits | -9.5 (-17.5, -1.5) | **0.020** | -0.0 (-7.6, 7.6) | 0.999 |
| Deep-fried food | 12.5 (3.5, 21.5) | **0.007** | 10.7 (2.5, 19.0) | **0.011** |
| Sweet desserts | 1.4 (-8.3, 11.1) | 0.775 | 3.7 (-5.5, 12.8) | 0.432 |
| Sugary beverages | -2.2 (-10.2, 5.7) | 0.582 | -0.8 (-8.1, 6.5) | 0.827 |
| Non-sugary beverages | -10.2 (-18.5, -1.8) | **0.018** | -11.7 (-19.6, -3.9) | **0.004** |
| Postmeal satiety | 8.1 (4.8, 11.3) | **<0.001** | 7.6 (4.6, 10.6) | **<0.001** |
| Postprandial light-intensity physical activity (hours) | -18.0 (-36.5, 0.6) | 0.058 | -24.8 (-42.2, -7.3) | **<0.001** |
| Postprandial moderate-to-vigorous-intensity physical activity (hours) | -68.4 (-88.6, -48.1) | **<0.001** | -55.9 (-74.8, -37.0) | **<0.001** |
| Daily light-intensity physical activity (hours) | -5.4 (-9.6, -1.2) | **0.011** | -7.6 (-11.3, -3.8) | **<0.001** |
| Daily moderate-to-vigorous-intensity physical activity (hours) | 0.0 (-5.9, 6.0) | 0.989 | 3.9 (-1.1, 8.9) | 0.124 |
| Sleep duration (hours) | -2.4 (-4.5, -0.3) | **0.024** | -3.0 (-5.0, -0.9) | **0.004** |
| Meal time of the day (reference: 06:00-12:00 hours) | | | | |
| 12:00-18:00 hours | 61.6 (54.5, 68.5) | **<0.001** | 57.4 (50.9, 63.7) | **<0.001** |
| 18:00-24:00 hours | 44.9 (37.5, 52.3) | **<0.001** | 45.0 (38.2, 51.7) | **<0.001** |

Estimates for the interaction terms with sex refer to additional effects in males compared with females (reference group). Estimates for interaction terms with prediabetes status refer to additional effects in those with prediabetes compared with those without prediabetes (reference group). The two models were adjusted for age, sex, ethnicity, education level, smoking, alcohol, body mass index, and mean 2-hour pre-prandial glucose level. The models were also mutually adjusted for meal time and within-person diet, physical activity, and sleep exposures, as well as their interaction terms with sex or prediabetes status. The unit of outcome postprandial glucose iAUC is mmol/l*minute. Postprandial measures were for the 2-hour window after eating. iAUC: incremental area under the curve.


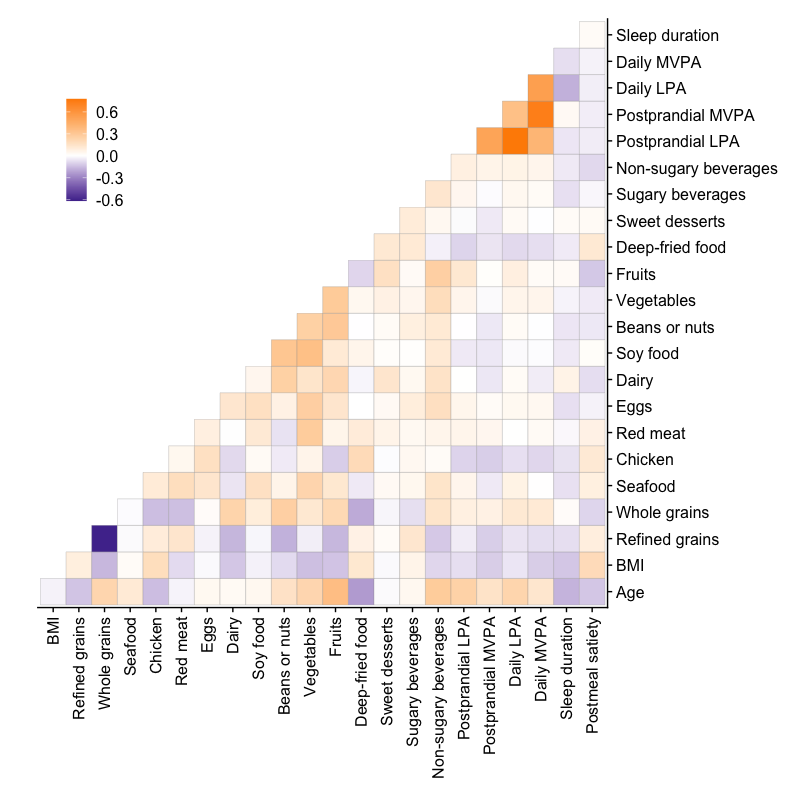


**eFigure 1**: Pearson correlation coefficient matrix for the numeric measures. The correlation coefficients were computed based on individuals’ average values for repeated measures. LPA: light-intensity physical activity; MVPA: moderate-to-vigorous-intensity physical activity.
